# Three-Dimensional Measurement and Analysis of Rod Deformation for Spinal Deformity Correction: A Novel Methodology

**DOI:** 10.3390/bioengineering13091012

**Published:** 2026-08-31

**Authors:** Sam W. Hendricks, Cale J. Hendricks, Arin M. Ellingson, David W. Polly, Arthur G. Erdman

**Affiliations:** 1 Department of Orthopedic Surgery, University of Minnesota, Minneapolis, 55454 MN, USA; swhend989@gmail.com (S.W.H.); ellin224@umn.edu (A.M.E.); pollydw@umn.edu (D.W.P.J.); 2 Department of Family Medicine and Community Health, University of Minnesota, Minneapolis, MN 55454, USA; 3 Department of Mechanical Engineering, University of Minnesota, Minneapolis, MN 55454, USA; agerdman@umn.edu

**Keywords:** posterior spinal fusion, rod contouring, deformity correction, three-dimensional analysis

## Abstract

The amount of rod contour is a critical factor that influences sagittal and coronal alignment for posterior spinal fusion. Distraction techniques to correct spinal deformity, which are often three-dimensional (3D) in nature, are known to cause rod deformation from the ideal, planned rod curvature. Therefore, surgeons often overbend rods to account for this phenomenon. Currently, there is no agreed-upon method to scientifically quantify rod unbending, and no methods offer a 3D analysis. The proposed methodology addresses this gap. By aligning the 3D rod centerlines, the segmented area enclosed between the two lines serves as a measure of rod deformation. This paper presents the foundational principles and concepts for quantifying 3D rod deformation. With further research and clinical implementation, this methodology has the potential to provide valuable surgical insight, which could improve postoperative spinal alignment and outcomes.

## 1. Introduction

The number of spinal fusion surgeries has increased significantly in recent years, driven by advances in surgical techniques and the development of innovative instrumentation [1,2,3,4]. Posterior spinal fusion has emerged as one of the most widely performed procedures for correcting spinal deformities, including adolescent idiopathic scoliosis (AIS) and adult spinal deformity (ASD), and has demonstrated strong clinical effectiveness [5,6,7,8,9]. Typically, these corrective procedures utilize bilateral longitudinal rods, which connect to posterior pedicle screws and serve as rigid structural elements for the application of corrective forces [10]. Rods are preoperatively contoured to achieve alignment goals [6]. Traditionally, these operations focused on correcting coronal plane alignment; however, recent attention has shifted toward the critical role of the sagittal plane [11,12].

Recent advancements in preoperative (pre-op) planning software, such as UNiD by Medtronic, allows surgeons to accurately engineer rod shape for specific patient applications and goals. However, the literature has shown significant rod deformation both intraoperatively after correction and postoperatively [6,7,8,13]. Currently, there is no way to preoperatively predict the degree of rod deformation, nor a standard way to measure it. Surgeons at our institution empirically overbend rods by 5–15% to address this deformation issue. While this approach offers a valuable foundation, further refinement is required to enhance pre-op prediction of rod deformation, given the inherent variability introduced by anatomical and biological differences among patients.

Previously, studies have employed various techniques to analyze two-dimensional (2D) rod deformation. One strategy involves comparing a pre-implantation paper rod tracing to a postoperative (post-op) X-ray, measuring maximal rod deflection and rod angle of curvature in a single plane [6,7,13]. Others have determined the rod’s plane of maximum curvature (RPMC) of a three-dimensional (3D) rod, where 2D deflection and implant rod angle of curvature were measured [8]. These techniques measure rod deformation that occurs in a single plane. However, a 3D analysis would give a more accurate measurement of rod deformation for these complex, 3D spinal deformities.

To the best of the authors’ knowledge, no studies have established a technique for measuring 3D rod deformation following posterior corrective spinal fusion. This paper presents a novel methodology aimed at addressing this gap by presenting an approach that could provide valuable clinical insight and improve post-op alignment. The method involves aligning pre-op and post-op rod profiles, where the defined area between rods represents the 3D magnitude of deformation. We will examine techniques to calculate this area, the associated challenges, and the applicability of the method in three dimensions. Ultimately, this approach has the potential to inform clinical decision-making and enhance pre-op planning for optimal alignment.

## 2. Concept/Technique Description

### 2.1. Generation of Rod Centerlines

Rod centerlines can be reconstructed from biplanar standing radiographs or CT and can be represented using Bezier or B-spline curves. CT is the gold standard for 3D reconstruction of metal hardware with minimal artifact or distortion; however, 3D reconstruction with biplanar radiographs has also been shown to be accurate [14,15]. The pre- and post-op rods can then be digitized along their entire centerlines by sampling points to capture their continuous three-dimensional geometry. The resulting centerlines can subsequently be resampled at uniform intervals along their arc lengths to establish corresponding points along the rods. Because the physical length of the rod theoretically remains constant (i.e., pre-op length = post-op length), corresponding points are located at equal physical distances along the rods. The two centerlines can then be rigidly registered by translating and rotating them in space to align corresponding points without altering their scale or curvature. In practice, reconstruction is performed with image-processing software such as 3D Slicer, Mimics, or Amira, while curve fitting, arc-length parameterization, and area calculations are implemented with concise custom code in MATLAB or Python.

### 2.2. Determination of 3-Dimensional Segmental Areas

**Figure 1:**
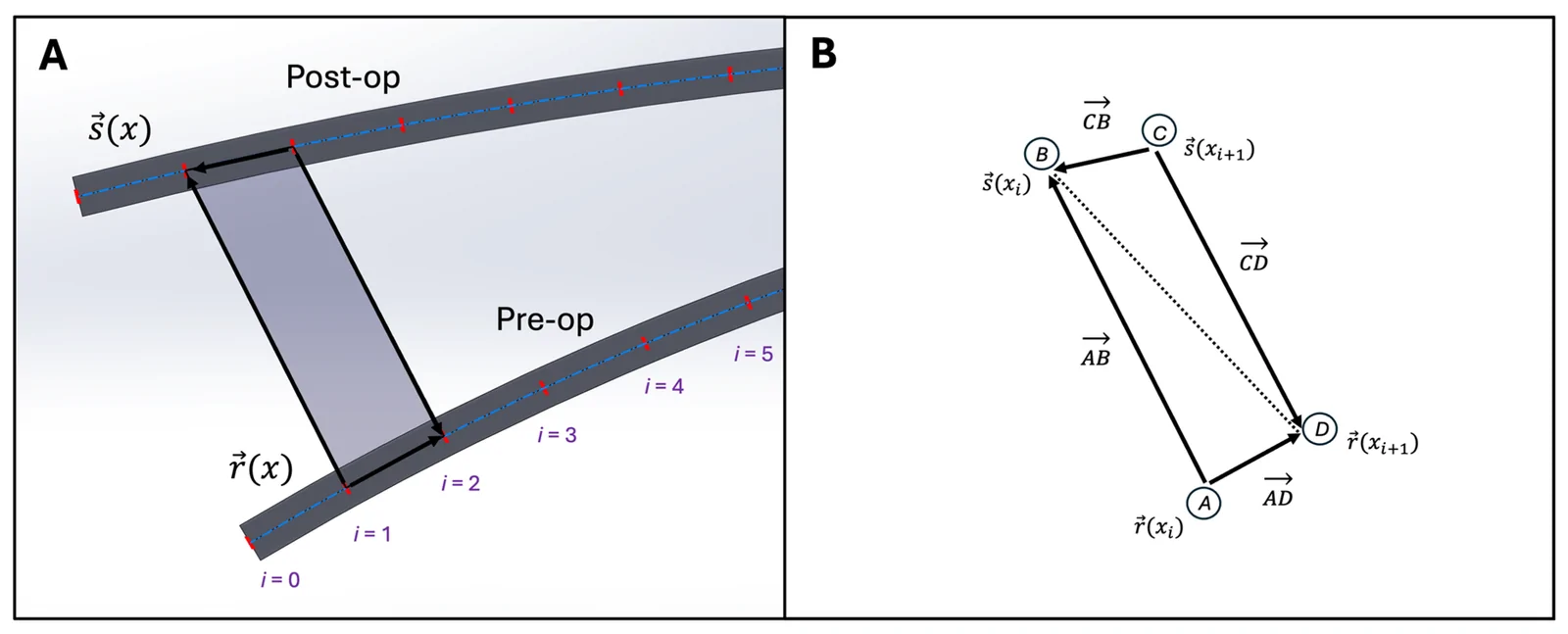
Schematic representation of the segmental area-of-deformation calculation. (**A**) 2D lateral display of the pre-op (bottom) and post-op (top) rod centerlines represented by blue dashed lines. The shaded region illustrates the area of deformation between corresponding rod segments, with red hash marks (points) denoting segmentation points indexed by *i*. (**B**) Vector representation of a quadrilateral formed between corresponding pre- and post-op points of a given segment. Points *A*, *B*, *C*, and *D* correspond to $\vec{r}(q_{i})$, $\vec{s}(q_{i})$, $\vec{s}(q_{i+1})$, and $\vec{r}(q_{i+1})$, respectively.

The pre-op rod represents the manufactured correcting rod measured before implantation, designed with a planned contour to achieve the desired sagittal alignment, while the post-op rod reflects the shape in vivo. This post-op time point is left to the researchers’ discretion, which may include intraoperatively after only the correcting rod is placed, immediately postoperatively, or at later time points allowing for sufficient physiologic mechanical loading. It is important to note that the correcting rod is the primary rod of interest. We assume that the centerlines of both rods have been accurately determined, as described above. To quantify differences, the rods are placed adjacent to each other (details on placement and orientation are discussed later in Section 2.4). The first step for area computation is to segment the rods with equally spaced points along the length of both the pre-op and post-op rods (Figure 1). For this methodology, we denote the function for the pre-op rod centerline as $\vec{r}(q)$, the post-op rod as $\vec{s}(q)$, where *q* is define as the length along a given rod, *n* as the total number of segments, and *i* as the index of the segmented points along the rods.

The bounded area is defined by two consecutive connecting lines linking corresponding segmentation points on the pre- and post-op rod centerlines (vectors $\vec{AB}$ & $\vec{CD}$), and the line segments connecting their endpoints along each rod (vectors $\vec{CB}$ & $\vec{AD}$). This quadrilateral represents the rod deformation for a given segment, as shown by the shaded region in Figure 1A.

**Figure 2:**
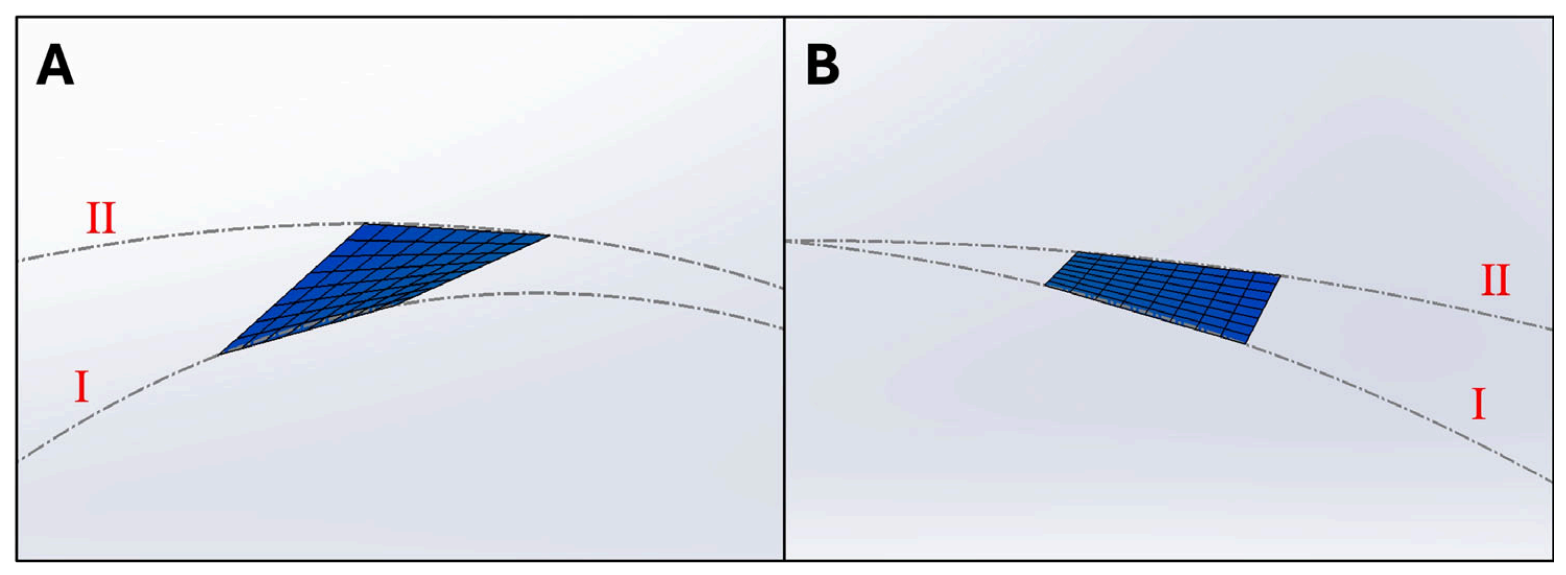
Illustration of a 3D skewed quadrilateral. Lines I and II represent non-parallel, non-coplanar curves. The blue surface is defined by segments that connect points on line I and line II. As lines I and II diverge in space, the resulting surface becomes twisted, forming a 3D skewed quadrilateral. (**A**,**B**) represent two different views of the same surface.

Given that the pre- and post-op rods may diverge in 3D space, the enclosed region may form a skewed quadrilateral that would not lie within a single plane (Figure 2A). Therefore, calculating the segmented area using planar triangular approximations provides a close and practical estimate without requiring more complex mathematical models. Each quadrilateral is divided into two triangles (Figure 1B), and the area of each triangle is determined using the cross product of two vectors sharing a common vertex. For two such vectors, the magnitude of their cross product is equal to the area of the parallelogram spanned by the vectors. Therefore, one-half of the cross-product magnitude gives the area of the corresponding triangle. Summing the areas of the two triangles yields the total area of the segmented quadrilateral, shown as follows.

(1) $$A_{i}=\frac{1}{2}\left[\left\|\vec{AB}\times \vec{AD}\right\|+\left\|\vec{CD}\times \vec{CB}\right\|\right]$$

Substituting Equation (1) with displacement vector equations (i.e., $\vec{AB}=B-A$) using the respective functions for the pre- and post-op rods, results in the following equation.

(2) $$A_{i}^{*}=\frac{1}{2}\left[\left\|\left(\vec{s}\left(q_{i}\right)-\vec{r}\left(q_{i}\right)\right)\times \left(\vec{r}\left(q_{i+1}\right)-\vec{r}\left(q_{i}\right)\right)\right\|+\left\|\left(\vec{r}\left(q_{i+1}\right)-\vec{s}\left(q_{i+1}\right)\right)\times \left(\vec{s}\left(q_{i}\right)-\vec{s}\left(q_{i+1}\right)\right)\right\|\right]$$

### 2.3. Determination of 3-Dimensional Total Area of Deformation

Extending Equation (2) for a single segmented quadrilateral over the entire length of the rods is accomplished by summing the absolute values of each segmented area along the length of the rod. This results in the following summation given a rod length (*L*), change in segment length ($∆q=q_{i+1}-q_{i}=\frac{L}{n}$), and the *ith* length ($q_{i}=i\frac{L}{n}$).

(3) $$A_{n}^{*}=\sum_{i=0}^{n-1}\frac{1}{2}\left[\left\|\left(\vec{s}\left(q_{i}\right)-\vec{r}\left(q_{i}\right)\right)\times \left(\vec{r}\left(q_{i+1}\right)-\vec{r}\left(q_{i}\right)\right)\right\|+\left\|\left(\vec{r}\left(q_{i+1}\right)-\vec{s}\left(q_{i+1}\right)\right)\times \left(\vec{s}\left(q_{i}\right)-\vec{s}\left(q_{i+1}\right)\right)\right\|\right]$$

**Figure 3:**
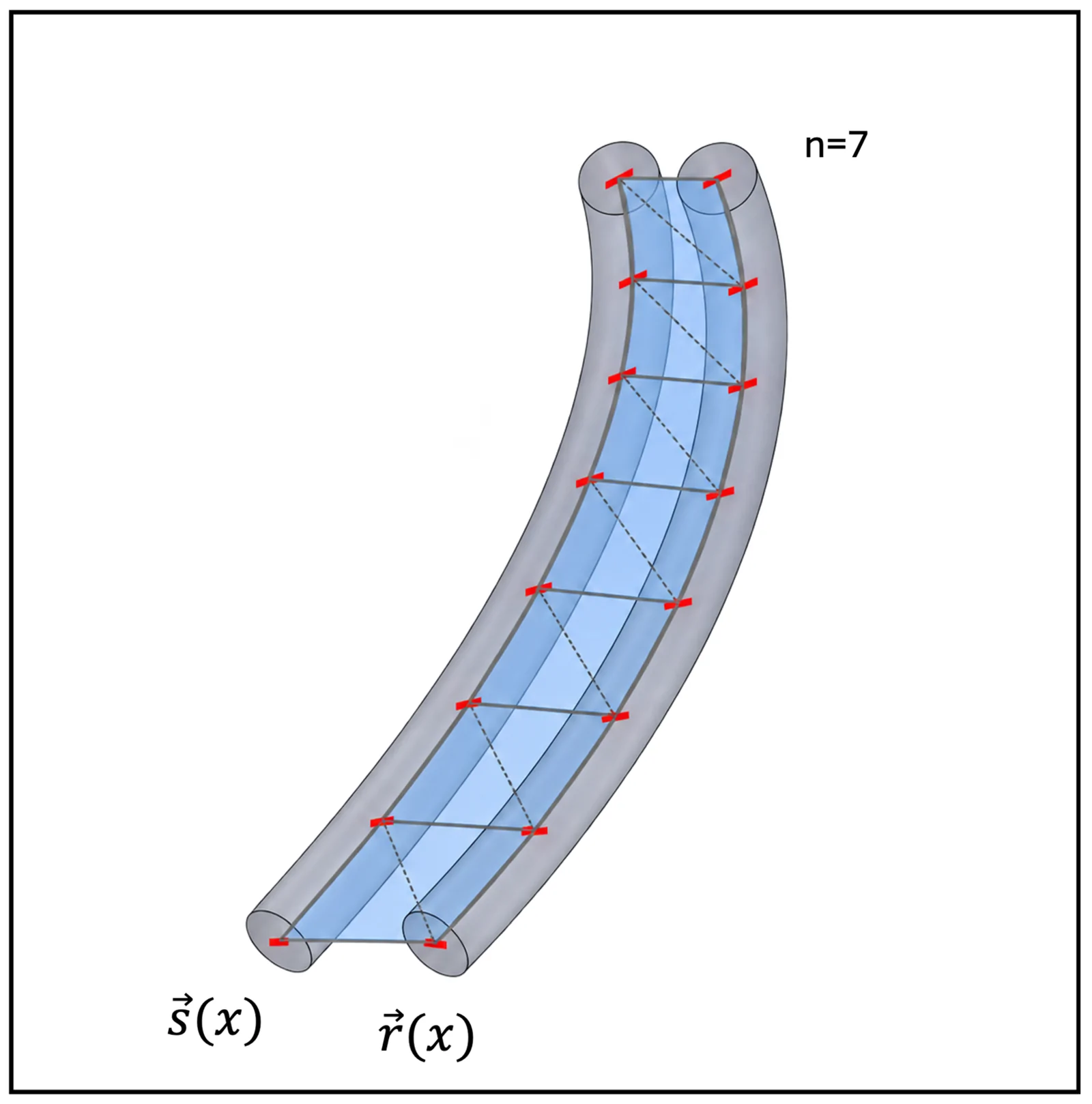
3D illustration of segmented areas between deflecting rods. The area of deformation is depicted by the blue shaded region, the red hash marks (points) represent rod segmentation, and the dark grey lines represent the centerlines of corresponding rods.

This summation, as depicted in Figure 3, can be evaluated numerically for various arbitrary *n* values using mathematical software such as MATLAB or Python.

To solve analytically, an integral equation from 0 to *L* can be derived using the limit definition of an integral.

(4) $$\int_{a}^{b}f\left(q\right)dq=\underset{n\to \infty }{\lim}\sum_{i=1}^{n}f(q_{i})∆q$$

First, the order of subtraction for the vectors in the second cross product of Equation (3) can be flipped, and the vectors between consecutive points on each rod centerline ($\vec{CB}$ & $\vec{AD}$) can be multiplied by $\frac{∆q}{∆q}$. As the number of segments increases, the segment length decreases (n→∞, ∆q≈0), allowing the defined vectors to be written as derivatives of the rod centerlines.

(5) $$\vec{r}\left(q_{i+1}\right)-\vec{r}\left(q_{i}\right)=\frac{\vec{r}\left(q_{i+1}\right)-\vec{r}(q_{i})}{\Delta q}\Delta q\approx {\vec{r}}^{\prime }\left(q_{i}\right)$$

(6) $$\vec{s}\left(q_{i+1}\right)-\vec{s}\left(q_{i}\right)=\frac{\vec{s}\left(q_{i+1}\right)-\vec{s}(q_{i})}{\Delta q}\Delta q\approx {\vec{s}}^{\prime }\left(q_{i+1}\right)$$

Here, Equation (5) is defined by left endpoints and Equation (6) by right endpoints. Furthermore, the summation can be distributed to each cross product in Equation (3). Given that $\sum_{i=0}^{n-1}f\left(q_{i+1}\right)=\sum_{i=1}^{n}f(q_{i})$ and the second cross product can be entirely written in terms of $q_{i+1}$, the bounds of the second summation can be adjusted accordingly. This results in the following limit equation.

(7) $$A_{limit}^{*}=\underset{n\to \infty }{\lim}\left[\sum_{i=0}^{n-1}\frac{1}{2}\left[\left\|\left(\vec{s}\left(q_{i}\right)-\vec{r}\left(q_{i}\right)\right)\times ({\vec{r}}^{\prime }(q_{i}))\right\|∆q\right]+\sum_{i=1}^{n}\frac{1}{2}\left[\left\|\left(\vec{s}\left(q_{i}\right)-\vec{r}\left(q_{i}\right)\right)\times \left({\vec{s}}^{\prime }\left(q_{i}\right)\right)\right\|∆q\right]\right]$$

Finally, using the definition of an integral (Equation (4)), we can transform Equation (7) into the definite integral as follows.

(8) $$A=\frac{1}{2}\int_{a}^{b}\left[\left\|\left(\vec{s}\left(q_{i}\right)-\vec{r}\left(q_{i}\right)\right)\times ({\vec{r}}^{\prime }(q_{i}))\right\|+\left\|\left(\vec{s}\left(q_{i}\right)-\vec{r}\left(q_{i}\right)\right)\times \left({\vec{s}}^{\prime }\left(q_{i}\right)\right)\right\|\right]dq$$

This equation represents the total area as the number of segments approaches infinity, reducing approximation error. As the length of each segment along the rods decreases, it more closely aligns with the true curvature of the rod centerline, and the triangular approximation more accurately represents the area between the two rod centerlines. Therefore, with a sufficiently large value of *n* for Equation (3), the summation provides a close approximation of the integrated area and may be sufficient in most practical applications.

As a result, the area of deformation could be used as a criterion for whether the pre-op rod successfully retained its shape within an acceptable value (maximum acceptable area). It is important to note that the total area of deformation could be divided by rod length to offer a proportional comparison between cases. This could be adapted to segmental area analysis as well. Additionally, this method allows for weight to be assigned to different parts of the rod, where each segment has a multiplying factor corresponding to its importance/significance. Locations on the rod where precision is more desirable, with respect to patient anatomy and/or rod shape, could be given a greater weight. The ability to customize different parts of the rod may be advantageous for certain spinal deformities.

### 2.4. Relative Rod Orientation

**Figure 4:**
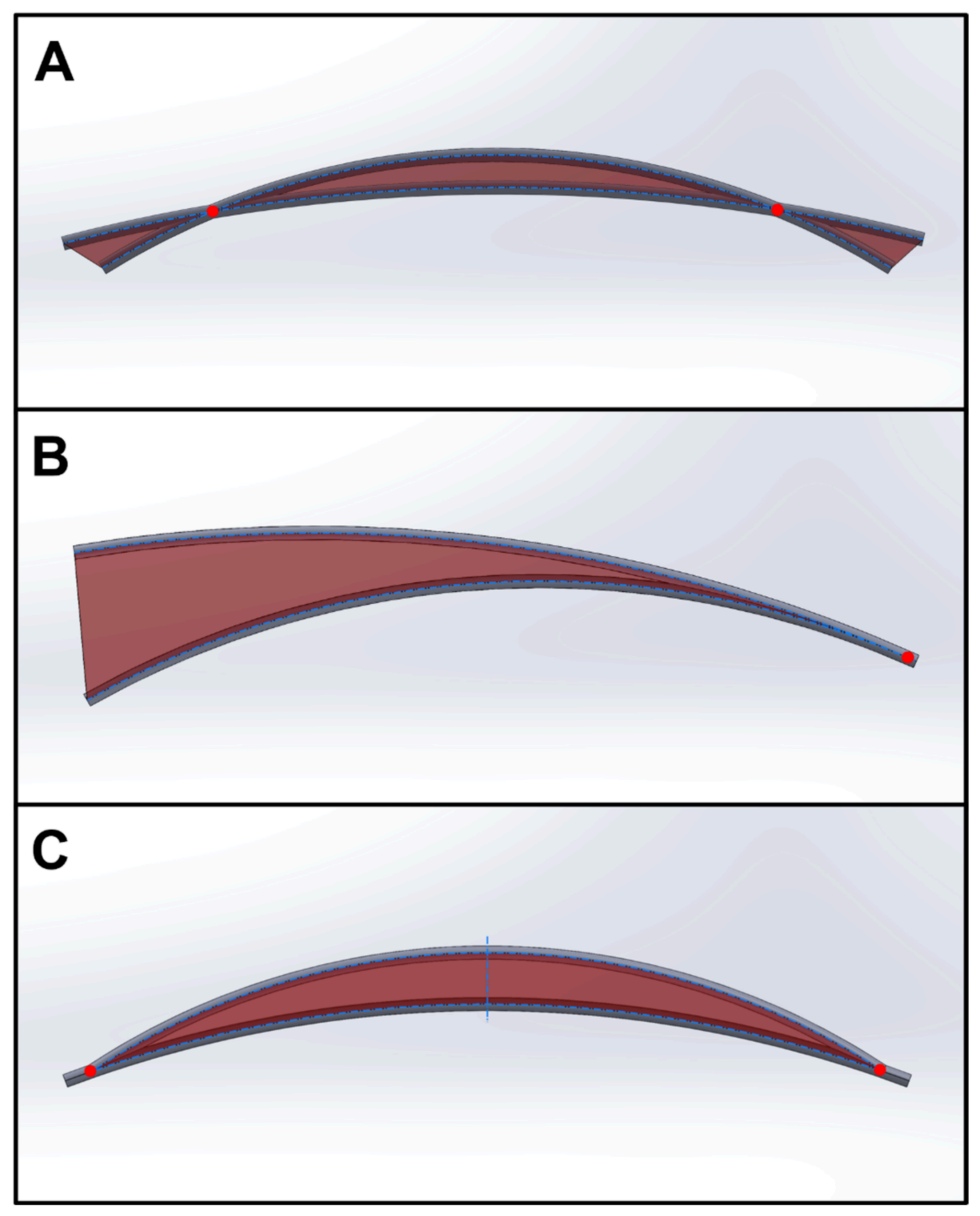
Orientations of pre- and post-op rods. The red region outlined represents the area of deformation for each respective method, the blue dashed lines represent corrresponding rod centerlines, and the red dots mark where the rod centerlines intersect. (**A**) Optimization of the total area of deformation. (**B**) Aligning caudal rod ends. (**C**) Minimizing area while centering the two rod ends with the more bent rod intersecting the less bent rod’s centerline.

Multiple ways of orienting rods with respect to one another have been considered. The first would be to minimize the overall area of deformation using curve optimization techniques (Figure 4A). The second could be to position the caudal ends of each rod at the same point with equivalent tangential lines. The less globally bent rod would lie above the more bent rod. This method would mimic the common cantilever distraction technique used for deformity correction (Figure 4B). The last method would be to minimize the total absolute area while centering the two rods respectively such that the endpoints of the more globally bent rod would lie along the less bent rod (Figure 4C). These approaches should be further investigated to determine which offers the most clinically accurate results.

The presented rod orientation can be applied at a global rod level or with respect to specific vertebral levels. The former can be achieved by mapping vertebral levels onto both the pre-op plan and post-op imaging, where distinct anatomical reference points, such as vertebral endplates, can define new rod endpoints. Therefore, a summation of segments bound within a defined rod section can offer a measure of deformation for that particular vertebral level. Opposed to taking the absolute value, as for the total area, directional deformation can be defined by assigning positive or negative values for areas respective to the concave or convex side of the pre-op rod.

## 3. Methodological Example

To further demonstrate this methodology outlined above, a numerical example is provided. Assuming spline equations for rod centerlines were previously found, we chose to align their caudal ends together (as shown in Figure 4B) for the sake of the example. We will use the following equations, where *q* is defined as the length of the rod.

(9) $$\vec{s}\left(q\right)=\left(q,\;0.015q^{2},\;0.0008q^{3}\right),\;0\leq q\leq 10$$

(10) $$\vec{r}\left(q\right)=\left(q,\;0.02q^{2},\;0.0015q^{3}\right),\;0\leq q\leq 10$$

**Figure 5:**
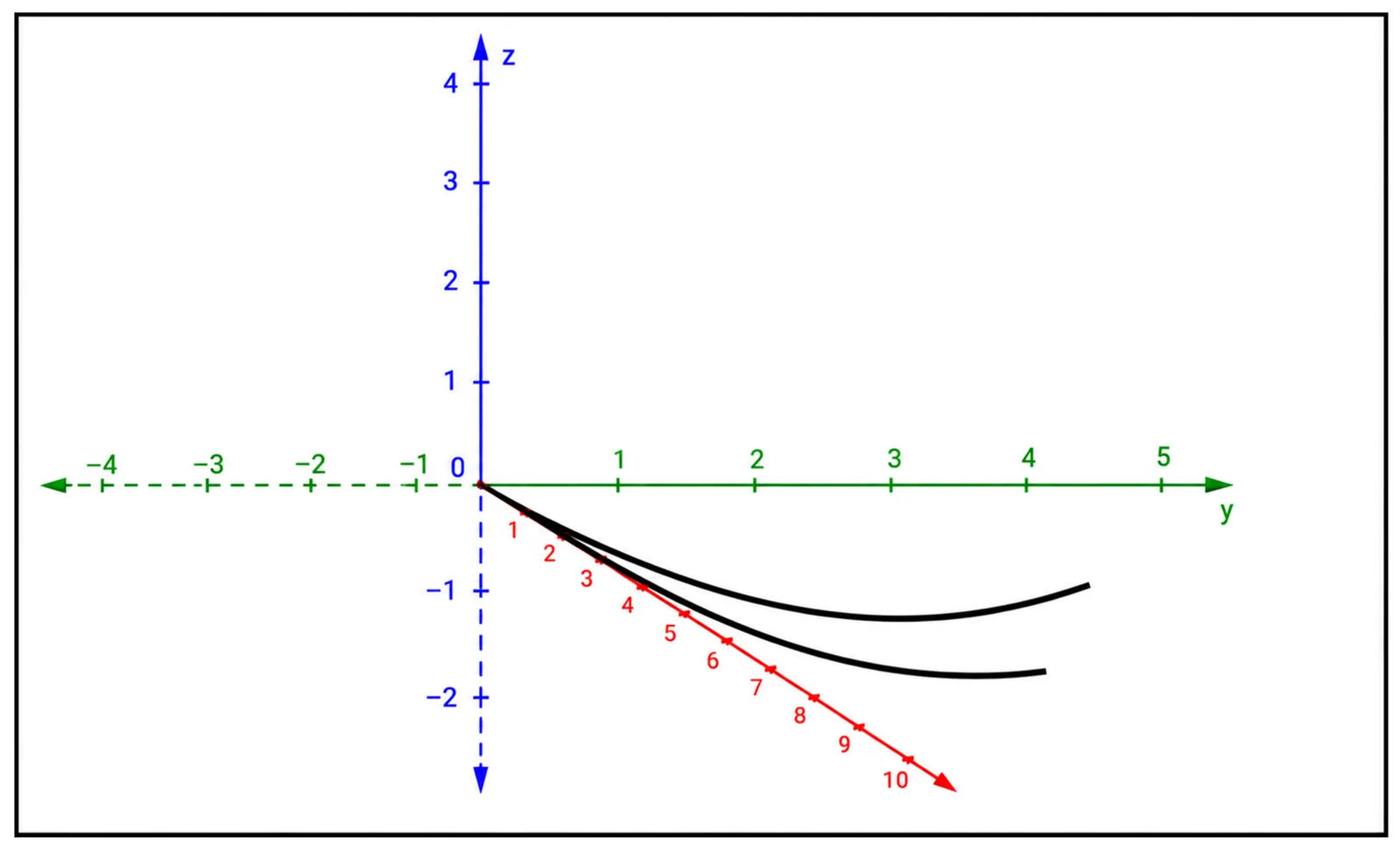
Graphical representation of spline equations. Axes for the *x*, *y*, and *z*, direction are shown with red, green, and blue arrows respectively. The two black curves represent the spine equations.

A graphical representation of the defined equations is shown in Figure 5.

A script was written in MATLAB (R2025a) to insert the above vector equations into the summation presented in Equation (3). Defining *n* values of 5, 10, 20, 50, and 100, the total areas were calculated as shown in Table 1.

The values in Table 1 demonstrate convergence to an area of ~2.445 in undefined units. Therefore, the integral area calculation, as defined by Equation (8), is not necessary for this example.

## 4. Discussion

Spinal deformity correction requires the achievement of an optimal sagittal and coronal alignment. Currently, the desired sagittal contour of rods is created in one of two ways: using pre-op modeling to manufacture patient-specific rods or manually bending rods intraoperatively [14,15]. However, rod deformation inevitably occurs as corrective forces are applied, and neither method properly accounts for rod deformation during implantation [15,16,17]. No current analytical tool exists to accurately predict the degree of rod over/under bending required to achieve the intended spinal alignment, and there are no universally agreed-upon methods to measure rod deformation [18]. The methodology presented in this paper aims to address this by providing a way to measure rod deformation in three dimensions.

In literature many different modalities for measuring rod deformation in two dimensions exists. Cidambi et al. analyzed sagittal rod deformation for patients with thoracic adolescent idiopathic scoliosis (AIS) using two different measurement techniques: maximum rod deflection, defined as the perpendicular distance from the line connecting rod endpoints, and maximum angle of rod curvature, defined as the angle subtended by the tangential lines drawn at the endpoints. The change in measurements was determined between an intraoperatively traced rod prior to implantation and a 5-week average post-op radiograph to determine deformation. Cidambi et al. found the rod deformation to be statistically significant (*p* < 0.05) using both measurement methods for concave rods in the sagittal plane. For instance, the concave rod had a decrease in deflection of 13 mm and a reduction in contour angle of 21° [6].

Sia et al. studied 21 AIS patients and performed a similar rod analysis to Cidambi et al. Deformation was quantified by measuring maximal rod deflection and the maximum angle of rod curvature. Over-contoured concave rods were traced prior to insertion and were compared to post-op lateral radiographs. Significant reductions (*p* < 0.05) in both angle and deflection were observed, with titanium rods showing an angle decrease of 23.5° and deflection decrease of 4.5 mm, while cobalt-chrome rods showed an angle decrease of 23° and deflection decrease of 12 mm, respectively [7].

Le Navéaux et al. analyzed the rod contour of 35 AIS patients. Maximum rod deflection and maximum angle of rod curvature were measured in the rod’s (2D) plane of maximal curvature (RPMC) determined from a 3D rod profile. Prior to insertion, 2D rod profiles were traced, digitized, and modeled with MATLAB. Following deformity correction, intraoperative radiographs were obtained, and specific points along the rods were identified to generate a 3D reconstruction [19]. Another 3D reconstruction was made from a biplanar slot scan, one week post-op Similar to Cidambi and Sia, the rod deformation was statistically significant (*p* < 0.05) for both the deflection and angle of curvature for the concave rods. The concave rod curvature and deflection decreased by an average of 21° ± 9° and 13 ± 7 mm, respectively, and remained unchanged following surgery [8].

All studies demonstrate a substantial amount of rod deformation following deformity correction with posterior spinal fusion. A recurring theme across these studies and others is the use of planar 2D measurements, particularly maximum contour angles and deflection distances. However, these metrics fail to measure 3D and segmental deformation.

Although 2D analysis remains the standard for measuring and classifying AIS, research has highlighted the distinct 3D nature of the condition, indicating that 2D measurements cannot fully capture the true curvature of the spine [20,21]. Pasha et al. utilized 3D models to reconstruct spinal centerlines and quantify two key parameters: writhe(coiling) and torsion (twist) [22]. Their findings revealed that more than half of the patients exhibited high writhe with low torsion, underscoring the complex 3D characteristics of scoliotic spines. These two geometric parameters were complementary yet weakly correlated, reflecting significant geometric variability between patients. A deeper understanding of AIS as a 3D deformity underscores the need for advanced 3D models to evaluate the rod-deforming forces experienced during correction accurately.

The measurement model presented in this paper aims to address this challenge with the goal of establishing a pathway toward a universally applicable method for assessing rod deformation in 3D. Following proper biomechanical validation, application of this methodology to a large patient cohort could enable us to estimate the amount of case-specific rod deformation. This, in turn, has the potential to improve physicians’ accuracy in achieving their goal alignment.

### Limitations

Several aspects of the presented methodology require further investigation. First, the methods presented quantify rod deformation based on the centerline bending of the rods but do not account for torsional rotation of the rod, as a twist in the rod may not alter centerline geometry. Tracking and recording torsional rotation of a smooth rod is challenging. Typically, a surgeon will use a strategy of trying to hold the rod fixed in the sagittal plane. A more precise assessment would require radiopaque markers or other identifying marks to quantify torsion explicitly, though we anticipate that ongoing technological advancements will help overcome this limitation. Second, this methodology assumes the pre- and post-op rods to be the same length; therefore, small differences in rod length due to biomechanical forces could affect results. Additionally, inherent errors exist in 3D reconstruction with both CT or bi-planar imaging, center-line extraction, equation fitting, etc. Therefore, biomechanical validation is required before clinical implementation. Lastly, the methodology must be adaptable and allow for adjustments when applied in a clinical setting. While this paper introduces the measurement framework, clinical validation with a large cohort is necessary.

## 5. Conclusions

Establishing the ideal rod contour during pre-op planning is essential for achieving optimal sagittal and coronal alignment. This study introduces a novel methodology for 3D measurement of rod deformation. By aligning rod centerlines and quantifying the area enclosed between them, this approach provides a potentially useful metric for deformation assessment. The concepts and principles presented here form the foundation for future work, such that assessment of a large, diverse patient cohort could enhance predictive accuracy in surgical planning and improve patient outcomes.

## Figures and Tables

**Table 1:** Example area calculations for various *n* values.

Number of Segments (*n*)	Total Absolute Area
5	2.5216
10	2.4640
20	2.4497
50	2.4457
100	2.4450

## Data Availability

No new data were created or analyzed in this study.
